# Differential association of cerebral blood flow and anisocytosis in APOE ε4 carriers at midlife

**DOI:** 10.1177/0271678X231173587

**Published:** 2023-05-03

**Authors:** Maria-Eleni Dounavi, Elijah Mak, Peter Swann, Audrey Low, Graciela Muniz-Terrera, Anna McKeever, Marianna Pope, Guy B Williams, Katie Wells, Brian Lawlor, Lorina Naci, Paresh Malhotra, Clare Mackay, Ivan Koychev, Karen Ritchie, Li Su, Craig W Ritchie, John T O’Brien

**Affiliations:** 1 Department of Psychiatry, 2152 School of Clinical Medicine, University of Cambridge, Cambridge, UK; 2 Centre for Dementia Prevention, University of Edinburgh, Edinburgh, UK; 3 Department of Clinical Neurosciences and Wolfson Brain Imaging Centre, University of Cambridge, Cambridge, UK; 4 Institute of Neuroscience, 8809 Trinity College Dublin, University of Dublin, Dublin, Ireland; 5 Division of Brain Science, Imperial College Healthcare NHS Trust, UK; 6 Department of Psychiatry, University of Oxford, Oxford, UK; 7 INM, Univ Montpellier, INSERM, Montpellier, France; 8 Department of Neuroscience, University of Sheffield, Sheffield, UK

**Keywords:** CBF, hemodynamics, APOE4, perfusion, RDW

## Abstract

Cerebral hemodynamic alterations have been observed in apolipoprotein ε4 (APOE4) carriers at midlife, however the physiological underpinnings of this observation are poorly understood. Our goal was to investigate cerebral blood flow (CBF) and its spatial coefficient of variation (CoV) in relation to APOE4 and a measure of erythrocyte anisocytosis (red blood cell distribution width – RDW) in a middle-aged cohort. Data from 563 participants in the PREVENT-Dementia study scanned with 3 T MRI cross-sectionally were analysed. Voxel-wise and region-of-interest analyses within nine vascular regions were run to detect areas of altered perfusion. Within the vascular regions, interaction terms between APOE4 and RDW in predicting CBF were examined. Areas of hyperperfusion in APOE4 carriers were detected mainly in frontotemporal regions. The APOE4 allele differentially moderated the association between RDW and CBF, an association which was more prominent in the distal vascular territories (p – [0.01, 0.05]). The CoV was not different between the considered groups. We provide novel evidence that in midlife, RDW and CBF are differentially associated in APOE4 carriers and non-carriers. This association is consistent with a differential hemodynamic response to hematological alterations in APOE4 carriers.

## Introduction

The brain relies on a constant supply of energy and nutrients via the bloodstream to function properly. Lack of energy reserves implies that even small perturbations for a short period of time in the blood supply can have devastating consequences. Brain perfusion, typically referred to as cerebral blood flow (CBF), is tightly regulated via several mechanisms such as cerebral autoregulation and neurovascular coupling.^1^ In Alzheimer’s disease (AD), it is well-established that extensive areas of hypoperfusion exist in the brain ^2–4^ with some contradictory evidence suggesting localized areas of hyperperfusion.^5^ In the preclinical disease stage, covering the period before any cognitive symptomatology in carriers of risk genes or with dementia family history, findings are discordant with reports of hyper^6–8^ and hypoperfusion.^9,10^ Methodological and sample size limitations could explain the variable results; however, an inverted U-shaped pattern in how perfusion changes has also been suggested. This pattern postulates that the well-established hypoperfusion in AD is preceded by hyperperfusion many years before dementia onset.^11^

In a recent data-driven study based on the ADNI cohort (mean age in the specific study ∼73 years old), hypoperfusion was described as one of the earliest changes in the brain in the AD trajectory, occurring earlier than amyloidosis.^12^ Before targeting this lower perfusion in people at early disease stages or at risk of dementia though, the pattern of observed hyperperfusion at earlier stages, if conclusively established, warrants further investigation. With a need for early disease biomarkers a deeper understanding of the underpinnings of this increased perfusion at midlife is needed. This pattern has been observed in carriers of the apolipoprotein ε4 (APOE4) allele^7,8,13,14^ which is the most well-known genetic risk factor for sporadic AD conferring a 4- fold (single copy) to 8–12 fold (two copies) increase in risk for future AD,^15,16^ as well as in people with subtle cognitive decline.^17^

A plausible explanation for the observed hyperperfusion is antagonistic pleiotropy with beneficial effects of the APOE4 allele in young and middle-aged populations (both in terms of cognition and cerebral physiology) and detrimental effects later in life. This is supported by evidence from cognitive studies showing better performance in carriers of the ε4 allele^18–20^ as well as by observations of higher cortical thickness in APOE4 carriers.^21,22^ However, the observed higher arterial spin labeling (ASL) signal, typically interpreted as hyperperfusion, if measured with ASL sequences, could also relate to intravascular signal contamination and arterial transit time delays.^23,24^ A further possibility, not necessarily excluding other explanations is tissue hypoxia, which can trigger compensatory hyperperfusion to counteract underlying pathological changes.^25^

Such pathological changes could potentially relate to blood properties, specifically to alterations occurring in the red blood cells (RBCs). RBCs have been shown to demonstrate morphological alterations and reduced deformability in AD and blood viscosity has also been found to be altered.^26–28^ Erythrocytes exposed to β-amyloid fibrils appear to be more elongated and less deformable compared to control conditions.^29^ Furthermore, the red blood cell distribution width (RDW), capturing variability in the size and shape of erythrocytes has been shown to be increased in AD and to increase the odds of future dementia.^30^ It is further associated with increased mortality and incidence of cardio and cerebrovascular disease ^31–33^ and it has been shown that it was causally associated with cognitive changes and dementia.^34^ RDW is a hematological variable which is not considered in studies of CBF in health and disease. In some studies, hematocrit levels are taken into account,^35^ however, this does not capture the variability of RBC sizes. RDW has rarely been evaluated in tandem with brain imaging measures. Studies investigating the association of RDW to white matter hyperintensities (WMH) have reported positive associations to periventricular WMH ^36^ and a more severe WMH burden.^37^ There are no studies to date examining how the variability in erythrocyte shapes and sizes impacts typically investigated physiological and functional indices such as CBF. Hence further examination of hematological variables such as RDW and their relation to CBF in APOE4 carriers could be revealing in terms of determining the mechanism and consequences of perfusion changes in those at risk of future cognitive decline and dementia.

In the present study our aim was to investigate how CBF varies with APOE4 status and how it relates to arterial transit time delays and RDW. In a subsample of participants from a pilot of the PREVENT-Dementia study, we have shown hyperperfusion in APOE4 carriers.^7,13^ Hence our hypotheses for the current study were that in the whole PREVENT-Dementia cohort we would observe areas of hyperperfusion in APOE4 carriers and that the regional coefficient of variation of the ASL signal, which is a proxy of arterial transit time delays^23^ will differ between APOE4 carriers and non-carriers. Furthermore, we aimed to determine how perfusion parameters were connected to the RDW and if this relationship was differentially modulated by APOE4 carriership.

## Materials and methods

### Cohort

Data from four study sites from the PREVENT-Dementia cohort were used (West London, Edinburgh, Cambridge and Oxford). Participants were recruited from multiple sources, with the recruitment goal being a sample of 50% people with and 50% without family history of parental dementia and an age range between 40-59 years old. An additional inclusion criterion for the study was the ability to complete written and verbal assessments in English. Participants were identified from sources including local dementia register databases, via the Join Dementia Research register (https://www.joindementiaresearch.nihr.ac.uk/), or by registering their interest through the PREVENT-Dementia website (https://preventdementia.co.uk/) and public presentations and engagement sessions as well as word of mouth. The study was approved by the London-Camberwell St Giles National Health Service Ethics Committee (REC reference: 12/LO/1023) which operates according to the Helsinki Declaration of 1975 (and as revised in 1983). All participants provided written informed consent. During their visit, clinical as well as imaging information was collected. All participants were deemed cognitively normal following physical, neurological and cognitive examination by clinicians; none had signs, symptoms or a formal diagnosis of mild cognitive impairment or dementia. Exclusion criteria for the study were a diagnosis of MCI or dementia and known MRI contraindications. More details on the study population can be found in Ritchie et al. 2012 and Ritchie et al. 2013.^38,39^

## MRI protocol

Overall, 563 ASL scans were acquired on 3 T Siemens (Siemens Healthcare, Erlangen, Germany) scanners with the following models: Prisma (Oxford, Edinburgh), Prisma fit (Cambridge), Verio (West London, Edinburgh) and Skyra (Edinburgh). As part of the PREVENT-Dementia protocol a range of MRI sequences were collected; the following acquisitions were used in the present study: a) T1-weighted Magnetization prepared rapid gradient echo (MPRAGE) – repetition time (TR) = 2300 ms, echo time (TE) = 2.98 ms, 160 slices, flip angle = 9°, voxel size = 1x1x1mm^3^ and b) ASL PICORE Q2TIPS – 50 pairs of control/tagged and one calibration image, TR = 2.5 s, TE = 11 ms, 14 slices, inversion time = 1.8 s, bolus duration = 700 ms or 1675 ms, flip angle = 90^°^, voxel size 3.0x3.0x6.0 mm. The ASL bolus duration was changed during the study and kept consistent following this initial change. 225 participants were scanned with bolus = 700 ms (two study sites) and 338 with bolus = 1675 ms (three study sites).

## Clinical evaluation

Blood samples were collected in EDTA tubes for standard full blood count and APOE genotyping. APOE genotype analysis was conducted on QuantStudio12K Flex to establish APOE variants. Information on the RDW was recorded based on full blood count reports. Three out of the four sites recorded the RDW-coefficient of variation (RDW-CV) which will be referred to henceforth as RDW using the following analysers: Siemens ADVIA 2120i (Siemens Healthcare, Erlangen, Germany), Sysmex XN10 FBC, Sysmex XE 2100 (Sysmex, Kobe, Japan) and the fourth site recorded RDW-standard deviation (RDW-SD distribution width at 20% frequency level) using a Sysmex XE-5000 analyser. Along with this, information on hematocrit and the mean corpuscular volume (MCV) was retained for analysis. The CAIDE (Cardiovascular Risk Factors, Aging and Dementia) score, capturing lifestyle risk for future dementia was calculated based on age, sex, education years, body mass index, blood pressure, exercise patterns and cholesterol.^40^

## MR image processing

### T1-weighted

The structural scans were segmented into gray matter (GM), white matter (WM) and cerebrospinal fluid (CSF) using the Computational Anatomy Toolbox – CAT12.^41^ A correction for WMH was applied whereby WMH were assigned to the WM class by the algorithm. Segmented GM and WM maps were retained for partial volume correction (PVC) in the T1 space. A brain mask was derived by adding up WM, GM and CSF tissue classes. GM masks with extensive dura within the GM mask were manually corrected with ITK-SNAP.^42^ A vascular territory atlas in MNI space with nine segmented vascular territories,^43^ the proximal, middle and distal territories of the anterior middle and posterior cerebral arteries (ACA, MCA, PCA) was registered to the individual T1-space per subject using nearest neighbor interpolation in CAT12.

### Arterial spin labeling

ASL data were processed using FSL’s BASIL toolbox.^44^ A preliminary analysis was run by supplying to *oxford_asl* solely the ASL and structural scans to capitalize on the boundary-based registration routine it employs. The registration from ASL to the structural space was retained and inverted. The analysis was subsequently rerun by supplying additional parameters along with PV maps to *oxford_asl*. As part of the default BASIL processing, motion correction, a vascular compartment correction and spatially adaptive data priors were applied to the data, which were also calibrated based on an M0 image.^45^ A single compartment model was used and the ASL white paper recommendations for CBF quantification were followed.^46^ The segmented CAT12 volumes were supplied to the FSL algorithm for further processing. GM and WM PV maps were registered to the ASL space using the inverted transform and spline interpolation. All registrations were visually assessed and in case of imperfections, linear registration (FLIRT) was used as an alternative method. The ASL data were visually inspected for potential artifacts. Participants with extremely low mean GM CBF <20 ml/100g/min were excluded from CBF analysis since this is an abnormally low value for a healthy participant^47^ and was attributed to acquisition/labeling imperfections. The ASL technique is single time-point, hence there is a possibility for intravascular signal contamination. A voxel-wise threshold of 120 ml/100g/min was applied for the CBF region-of-interest (ROI) analysis. PVC is an essential step to ensure that neurovascular deficits are not masked by underlying atrophy, hence the analysis was run with PVC in native ASL space.^48,49^ Along with the GM and WM maps, individual vascular territory atlases were also registered to the ASL space using the generated registrations detailed in the previous steps and nearest neighbor interpolation. The ROI analysis within the vascular territories was confined in voxels with more than 50% of GM content and was run in the native ASL space.

**Figure 1.**
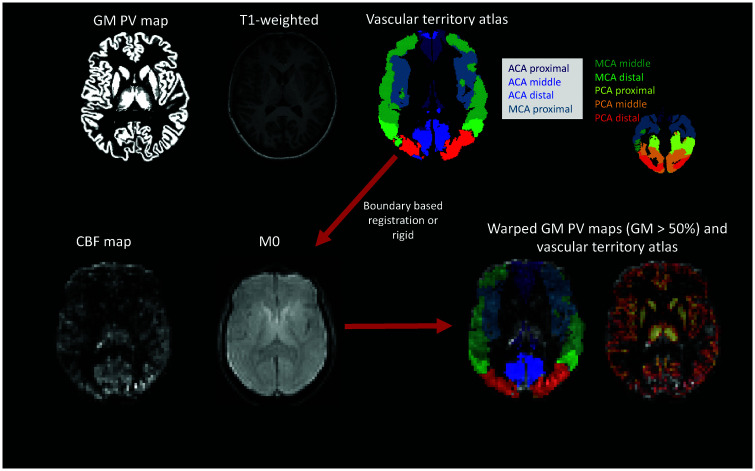
Analysis pipeline overview. The T1-weighted scans were segmented using CAT12 and gray matter (GM) and white matter (WM) partial volume maps were retained. A vascular territory atlas was also referred to each subject’s native space. T1-weighted scans were registered to the M0 acquisition for the ASL data. A boundary-based registration was used and alternatively rigid registration. In the ASL space the GM PV maps were thresholded to a level of 50% to retain only voxels with more than 50% GM. This map was also multiplied with the registered vascular territory maps. The GM maps were registered to the ASL space using spline interpolation and the vascular territory atlas using nearest neighbour interpolation. ACA: anterior cerebral artery; CBF: cerebral blood flow; GM -gray matter; MCA: middle cerebral artery; PCA: posterior cerebral artery; PV -partial volume.

Single time-point ASL without applied crusher gradients to suppress intravascular fast-moving spins is sensitive to intravascular signal contamination. Hence, we have further measured the spatial coefficient of variation (CoV) of the GM CBF using the formula: CoV= σ/μ which has been shown to relate to arterial transit time – ATT.^23^ This analysis was run without excluding voxels with CBF more than 120 ml/100g/min or artifacts related to arterial transit time or labeling asymmetries. Example CoV values and CBF maps are shown in Supplementaty Figure 1. An overview of the analysis pipeline can be found in Figure 1.

## Statistical analysis

Demographic variable and ROI analysis was conducted using MATLAB 2021 b (MathWorks Inc, Natick, Massachusetts, USA). Demographic factors were compared between APOE4 carriers (carriers of at least one APOE4 copy; including ε2/ε4) and non-carriers using the Wilcoxon rank sum test for continuous variables and the χ^2^ test for categorical variables. CBF and CoV data from the nine examined ROIs were harmonized between sites and different bolus durations (only one site had mixed boli) using the COMBAT harmonization algorithm^50^ with age, sex, years of education, dementia family history and APOE4 status as modulators. In case where participants were missing modulator variables they were excluded from the analysis; this applied to four participants for the CBF analysis who were missing information on APOE4 genotype and five for the CoV analysis, one was missing information on education and the rest for APOE4. Robust linear regression models with age, sex, years of education and APOE4 genotype were run.

To examine how APOE4 interacts with RDW to predict perfusion, robust linear regression models with APOE4*RDW interactions were used with further predictors in the models age, sex and years of education. For each investigated association the false discovery rate (FDR) multiple comparison correction method was applied (over the nine examined vascular territories). To investigate whether observed effects would generalize to other hematological parameters, we further examined interaction effects of APOE4 with hematocrit and MCV.

**Figure 2.**
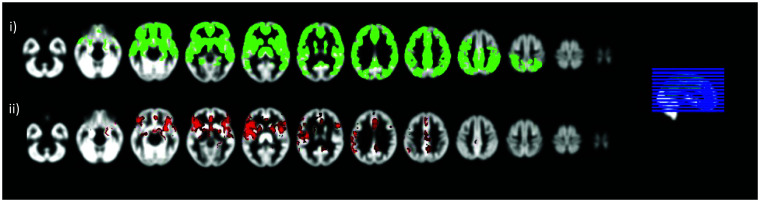
Voxel-wise analysis of blood flow differences between APOE4 carriers and non-carriers. i) the mask used for voxel-wise analysis, only the green voxels are included and ii) voxels whereby APOE4 carriers have significantly higher perfusion compared to non-carriers shown with red.

CBF values were compared between the groups at a voxel-wise level. CBF and PV GM maps were registered to the MNI space using DARTEL^51^ and a final voxel resolution of 2 mm. Voxel-wise analysis was run to investigate CBF differences between APOE4 carriers and non-carriers. Due to partial brain coverage, the field-of-view (FOV) largely varied between and within sites. A study-specific mask was constructed and was confined to voxels where the PV GM CBF was more than 4 ml/100g/min and less than 150 ml/100g/min for all participants. The mask was multiplied with a GM mask constructed based on the group-average GM within the voxel and thresholded at GM >25% (Figure 2). Voxel-wise COMBAT harmonization of CBF values within the GM mask was run with five centers (reflecting both center and bolus differences) and age, sex, APOE4 and years of education as modulators. Analysis was run with FSL’s *randomise,*^52^ with age, sex and years of education as covariates. All covariates were mean-centered, the threshold-free cluster enhancement (TFCE) method was used and 5000 permutations were applied; the level of significance was set to p < = 0.05.

A set of further exploratory and sensitivity analyses were run: a) to investigate if a higher CoV was associated with altered perfusion, regional CoV was added to the robust linear regression models predicting CBF, b) sex- specific differences in hemodynamics were investigating by including APOE4 * sex in the models c) Pearson correlations were run between RDW and regional CBF and CoV, d) voxel-wise APOE4 * RDW interactions in predicting CBF were investigated, e) the ROI CBF analysis was repeated after excluding ε2/ε4 carriers, f) the ROI CBF analysis was run for a three-group comparison (APOE4 non-carriers, heterozygotes and homozygotes), g) the hematocrit was further included in the models investigating APOE4 * RDW interactions to examine whether any APOE4 * RDW effects would persist when accounting for hematocrit and h) systolic blood pressure (SBP), cholesterol and hyperlipidemia status were added in the models investigating APOE4 * RDW interactions to examine if any observed associations would persist following inclusion of cardiovascular risk factors in the models.

## Results

Demographic characteristics of the sample are shown on Table 1. Overall data from 514 participants were used for the CoV analysis and 375 for the CBF analysis. The APOE4 carrier group was significantly younger compared to the non-carriers and a higher number had hyperlipidemia. GM CBF was not associated with age (t = 1.53, p = 0.13) or sex (t_female_ = 1.42, p = 0.16). On the contrary, there was a significant positive association between age and GM CoV (t = 2.98, p < 0.01) and females tended to have a lower GM CoV (t_female_ = −6.97, p < 0.01). CAIDE was not associated with total GM CBF but it was associated with GM CoV (ρ = 0.16, p < 0.01). There were no differences in the normalized GM or WM volumes between the groups. Datasets were excluded from the CBF analysis if they were of bad quality, if they were affected by artifacts or incidental findings were present (63 datasets) or if the mean GM CBF following PVC was lower than 20 ml/100g/min (121 datasets).

**Table 1.** Demographic specifications of the analysable cohort.

	CBF analysis (375)			CoV analysis (514)		
	APOE4- (233)	APOE4+ (142)	p-val	APOE4- (317)	APOE4+ (197)	p-val
Age (years)	51.9 ± 5.4	50.4 ± 5.6	0.01	51.8 ± 5.3	50.6 ± 5.5	0.02
Sex (%female)	66.5	68.3	0.72	63.4	64.0	0.90
Education (years)	16.4 ± 3.4	16.9 ± 3.6	0.37	16.4 ± 3.3	16.8 ± 3.5	0.32
FHD (%)	51.9	62.7	0.04	46.4	61.9	<0.01
APOE4 Homozygotes	N/A	20	N/A	N/A	30	N/A
Hematocrit^a^	0.42 ± 0.04	0.41 ± 0.04	0.19	0.42 ± 0.04	0.42 ± 0.03	0.43
Hemoglobin^a^	13.9 ± 1.3	13.9 ± 1.1	0.77	14.0 ± 1.3	14.0 ± 1.1	0.79
RDW (%)^b^	13.4 ± 1.0	13.3 ± 0.9	0.57	13.4 ± 1.0	13.3 ± 0.9	0.31
MCV	90.2 ± 5.5	90.1 ± 5.0	0.84	89.5 ± 5.6	89.9 ± 5.4	0.40
SBP (mmHg)	123.8 ± 17.0	122.5 ± 14.3	0.42	124.9 ± 16.5	124.1 ± 14.3	0.68
Cholesterol	5.5 ± 1.0	5.6 ± 1.1	0.61	5.5 ± 0.9	5.6 ± 1.1	0.35
Hyperlipidemia (%)	6.0	16.2	<0.01	6.6	14.7	<0.01
CAIDE	4.92 ± 2.47	4.49 ± 2.57	0.08	4.94 ± 2.45	4.63 ± 2.61	0.16
Normalized GM volume (%)	0.45 ± 0.02	0.45 ± 0.02	0.32	0.45 ± 0.02	0.45 ± 0.02	0.25
Normalized WM volume (%)	0.35 ± 0.02	0.35 ± 0.02	0.94	0.36 ± 0.02	0.35 ± 0.02	0.60

Values are shown as mean ± standard deviation or as percentages. APOE4: apolipoprotein ε4; CAIDE: cardiovascular risk factors, aging and dementia; CBF: cerebral blood flow; CoV: coefficient of variation; FHD: family history of dementia; MCV: mean corpuscular volume; RDW: red blood cell distribution width; SBP: systolic blood pressure.

^a^5 Participants missing values for the CBF and 11 for the CoV analysis.

**^b^** 94 Participants without RDW-CV values for the CBF and 204 for the CoV analysis.

## APOE4 effect on CBF and CoV

**Figure 3.**
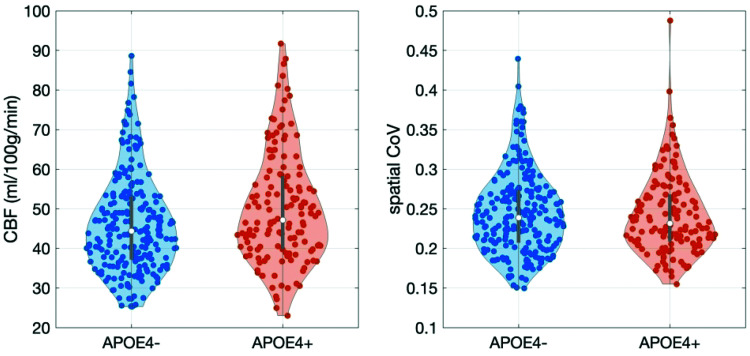
Violin plots of the CBF and CoV differences between APOE4 carriers and non-carriers within the significant cluster following voxel-wise analysis. CBF was significantly higher in APOE4 carriers in a model with age, sex, years of education and APOE4 as predictors. There were no differences between APOE4 carriers and non-carriers in the CoV for the significant cluster.

In voxel-wise analysis, a region largely covering the proximal MCA territory demonstrated hyperperfusion in APOE4 carriers. Clusters of hyperperfusion were also detected in the proximal ACA and middle MCA (Figure 2). Based on an overlay with an MNI structural atlas in FSLeyes, areas of difference following TFCE were mainly localized in the frontal and temporal lobes, cingulate, angular, paracingulate, precentral gyri and insula. Values within this identified cluster per group for CBF and CoV are shown on Figure 3. CBF was significantly higher in APOE4 carriers (t = 3.19, p < 0.01) and there was no difference in the CoV (t = −0.41, p = 0.68). In the ROI-based analysis within nine vascular territories (perfused by the internal carotid and the vertebral/basilar arteries – Figure 1), there was a trend towards higher perfusion in APOE4 carriers which did not remain significant following FDR and was mainly focused on the proximal MCA territory (t = 2.54, p = 0.01, p_FDR_ = 0.10). CoV was not different between APOE4 carriers and non-carriers for any of the considered ROIs. ROI analyses results are shown in more detail on Supplementary Table 1.

Associations between regional CBF and CoV for all territories but the proximal MCA (t = −1.49, p = 0.14) and middle MCA (t = −1.29, p = 0.20) were highly significant (p < 0.001); all associations were negative. There were no interactions between CoV and APOE4 in predicting CBF.

## APOE4 – RDW associations in predicting cerebral perfusion parameters

**Figure 4.**
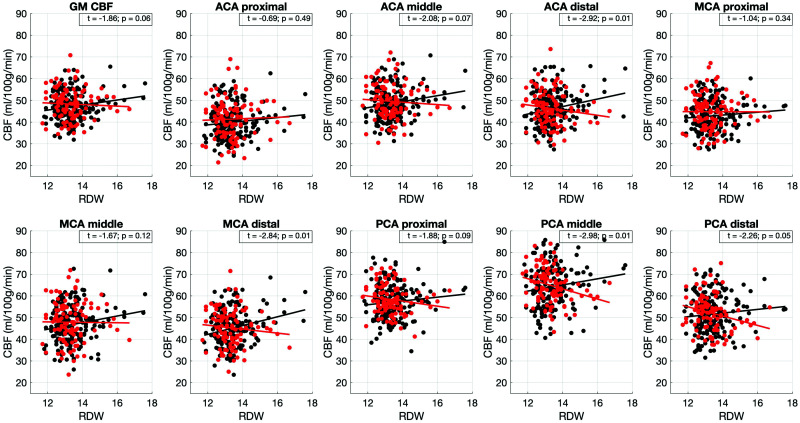
Associations between APOE4 and red blood cells distribution width in predicting blood flow. Red color is used for APOE4 carriers and black for non-carriers. The lines were fitted using first order polynomials. Shown values in individual boxes depict the t-score corresponding to the APOE4*RDW regression term in the applied linear regression models and p is the FDR corrected p-value for the association. ACA: anterior cerebral artery; CBF: cerebral blood flow; GM: gray matter; MCA: middle cerebral artery; PCA -posterior cerebral artery; RDW: red blood cell distribution width.

310 participants had available RDW (RDW-CV) data (281 CBF analysis). Correlations between RDW and CBF and CoV revealed one significant association between RDW and the CBF of the distal MCA (ρ = 0.12, p = 0.05) and no associations between regional CoV and RDW. We found several significant interactions between APOE4 and RDW when predicting CBF, whereby the directionality of the association was opposing between APOE4 carriers and non-carriers (Figure 4). Two associations were observed between APOE4 and RDW in predicting CoV, for the distal MCA (t = 1.96, p = 0.05, p_FDR_ = 0.19) and middle PCA (t = 2.08, p = 0.04, p_FDR_ = 0.19) which did not survive FDR. Voxel-wise analysis did not reveal significant clusters for the investigated interaction term. Within the APOE4 hyperperfusion cluster, the APOE4* RDW interaction term was not significant (t = −0.62; p = 0.54).

When hematocrit was further added to the linear regression models investigating APOE4 * RDW interactions the following significant APOE4 * RDW interactions persisted: distal ACA (t = −2.33, p = 0.02), distal MCA (t = −2.41, p = 0.02), middle PCA (t = −2.87, p < 0.01) and distal PCA (t = −2.01, p = 0.05), with the hematocrit being significantly negatively associated to CBF in all regions. Inclusion of SBP, cholesterol and hyperlipidemia status in the models did not change the observed associations. The interaction term remained significant, whereas cholesterol, SBP and hyperlipidemia status were not associated with the regional CBF in the presence of the interaction term in the distal ACA, MCA and PCA territories and the middle PCA.

## Sensitivity analysis

We repeated the ROI CBF analysis by excluding 11 ε2ε4 carriers in the cohort used for CBF analysis; results remained largely unchanged (significant RDW * APOE4 interactions following FDR: distal ACA – t = −2.81, p_FDR_ = 0.02; distal MCA – t = −2.69, p_FDR_ = 0.02; middle PCA t = −2.88, p_FDR_= 0.02; CBF in the proximal MCA was higher in APOE4 carriers t = 2.33, p = 0.02, p_FDR_ = 0.18). When APOE4 was considered as a three-group variable the same effect was detected for homozygotes and heterozygotes for the proximal MCA CBF (CBF significantly higher). This was not the case for the RDW interaction analysis for which the results were driven by APOE4 heterozygotes. There were no APOE4 * sex interactions when predicting regional CBF (t < 1.8, p > 0.07) or CoV (t < 1.06, p > 0.29).

## Discussion

Based on previous observations of hyperperfusion in middle–aged APOE4 carriers, we investigated associations between cerebral blood flow, arterial transit time variations and anisocytosis. There are several key findings in this study: a) we observed a pattern of higher perfusion in APOE4, b) we have shown that APOE4 changes the association between CBF and red blood cell distribution width such that APOE4 carriers demonstrate a negative association between RDW and CBF as opposed to non-carriers mainly in middle and distal vascular territories and c) using a proxy for ATT, the ASL spatial coefficient of variation, we have shown that this parameter was not significantly different between carriers and non-carriers and RDW did not interact with APOE4 in predicting the CoV. These findings imply that at this early asymptomatic stage, it is RDW and not the CoV (proxy for arterial transit variations) alterations that is differentially associated with cerebral perfusion between APOE4 carriers and non-carriers.

RDW, a measure routinely quantified as part of a regular hematological analysis capturing variations in the size and shape of erythrocytes, has not been considered in the past in studies investigating cerebral hemodynamics. It has been found that higher RDW levels are associated with cardiovascular disease, increased morbidity^33^ and increased odds of having dementia.^30^ RDW is associated with inflammation and it has been suggested that inflammation might actually promote increases in RDW by impacting erythropoiesis and RBC survival.^53^ It has also been shown that increases in RDW are connected to changes in erythrocyte deformability.^54^ RDW has also been found to relate to lower performance in cognitive tasks in middle-aged non-anaemic subjects.^55^ In a study investigating associations between erythrocytic properties and cognition it was found that MCV and RDW were causally associated with cognitive outcomes.^34^

RDW was not different between APOE4 carriers and non-carriers, however, its coupling with CBF and CoV was in opposing directions between the groups, especially in middle and distal territories. Considering that RBCs are the transporters of oxygen to the tissue, this observation could suggest a contribution from erythrocytes to neurovascular alterations observed during the preclinical and prodromal dementia stages. In the context of different AD hypotheses, the ‘erythrocytic hypothesis’ postulating that biochemical and morphological erythrocytic changes might play a role in the observed pathological cascade has been proposed.^56^ A higher RDW could be associated with co-existing inflammation and oxidative stress which can lead to impaired erythropoiesis and production of immature RBCs by the bone marrow.^53,57^ Tissue hypoxia has also been shown to affect hemorheological properties and is known to be present in AD.^58^

In the context of CBF as measured with ASL it is important to consider how a higher RDW would impact the observed signal and perfusion. ASL measures perfusion at the level of capillaries and the intensity of the signal could be impacted by multiple factors related to blood rheology or arterial transit time variations. More viscous blood would lead to slower flow and to observations of delayed or lower perfusion. A higher RDW could relate to reduced RBC deformability. Erythrocytes of different sizes also have varying hemoglobin contents, which could lead to inefficient tissue oxygenation.^59^ Furthermore, using in silico, in vivo and in vitro methods, higher RDW has been shown to relate to altered flow patterns and to increased interactions of blood cells with the arterial wall, potentially promoting atherosclerosis.^60^ Though RDW did not differ between APOE4 carriers and non-carriers, the observed interactions which were focused on middle and mainly distal territories (i.e. further from the feeding arteries, less well perfused^61^) could suggest a differential mechanistic response to underlying causes (for example inflammation or oxidative stress) of RDW increases. In these areas of difference, APOE4 carriers demonstrated a trend towards a negative association between CBF and RDW as opposed to non-carriers, an observation which could potentially suggest a successful underlying compensation in these regions for non-carriers. It needs to be noted that voxel-wise interaction analysis did not pick up significant clusters, an effect likely related to the limited FOV coverage. Within the cluster demonstrating hyperperfusion in APOE4 carriers (frontotemporal regions), the RDW * APOE4 interaction was not significant in predicting CBF. When a three-group variable was considered, the effect was shown to be driven by APOE4 heterozygotes. This observation could relate to inefficient power to detect an effect for homozygotes due to the small sample size (20 homozygotes).

Along with RDW, which is a measure of erythrocyte morphological variability, hematocrit and the MCV were also evaluated. A higher hematocrit was related to lower CBF. When interactions between hematocrit or MCV with APOE4 in predicting perfusion were examined, these were not significant. This suggests that it is specifically erythrocyte morphology and not their volume or proportion in the blood that drives the differential association with APOE4 in predicting brain perfusion. Studies investigating the effect of hematocrit on blood T1 relaxation time have shown that CBF can be mis-estimated if hematocrit is not taken into account.^35^ RDW has not been investigated yet in relation to blood T1; in light of our findings such an investigation could further inform our understanding of the observed associations.

Arterial transit time variations can greatly impact cerebral perfusion estimation and several techniques have been developed for their quantification.^62^ However, these intricate ASL schemes have not yet been incorporated in large study protocols and single time-point acquisitions such as PICORE used in the present study are very common. The proposed CoV as a surrogate ATT measure has shown promise and has been validated in relation to ATT derived from ATT-sensitive ASL sequences and to mean transit time from ^15^O PET.^23,63^ We did not find differences in CoV between APOE4 carriers and non-carriers. The CoV was significantly associated with CBF measurements. However, for the proximal MCA territory where hyperperfusion was recorded, this was not the case, suggesting that the quantified increased ASL signal might relate to actual hyperperfusion, though to establish this, an actual quantification of ATT would be more informative.

Hyperperfusion in the proximal MCA in APOE4 carriers could be observed due to the fact that ATT in the proximal MCA is relatively shorter compared to the other territories;^43^ it also had a better FOV coverage. In addition to this, the area covering the MCA territories, especially the proximal and middle ones was the area more prone to vascular signal contamination which was not entirely negated by the applied thresholds. When including the whole GM CoV as a covariate instead of the CoV within the proximal MCA, it was significantly associated with MCA CBF, the APOE4 effect also remained significant. RDW and CoV were also differentially associated between APOE4 carriers and non-carriers for some vascular territories, although the association was not significant following FDR. CoV is a relatively new index and it has been evaluated in a limited number of studies to date. In a cohort with MCI and early AD patients, it has been shown that CoV increased with cognitive status deterioration.^64^ In a population study where the prognostic capacity of CoV, CBF and WMH were investigated as predictors of cognitive decline, CoV and CBF did not predict changes in the MMSE score or people who would go on to develop dementia.^65^

Several sex-specific differences were detected for the examined hemodynamic measures. In particular, female participants demonstrated a lower CoV in all vascular territories and higher perfusion in the distal ROIs. It needs to be noted that the mean age of the PREVENT-Dementia cohort is 51 years old, hence female participants are at an age range where menopause effects (menopause is known to impact perfusion) are manifesting themselves.^66^ The consistent lower CoV in the brain in females which is indicative of a lower ATT, along with higher perfusion in the distal territories, might be related to cardiovascular risk, with males known to be at higher risk of cardiovascular disease and stroke.^67^ Indeed, the CAIDE score in the cohort was higher in males compared to females.

We did not observe age-related effects on global GM CBF and counter-intuitively for some ROIs the effect of age on CBF was positive, which is contrary to what is expected and reported in the literature in cohorts with larger age ranges.^68^ This could partly be explained by the equally positive association between the CoV and age in some of the territories and by the narrow age range of the cohort (40–59 years old). A sequence allowing for both quantification of CBF and ATT would be needed to disentangle the observed effect.

This is the first study to date investigating a measure of anisocytosis in relation to CBF in the AD trajectory and specifically examining the effect of APOE4 status by focusing on differences between carriers of at least one copy of APOE4 and non-carriers. We found evidence of a differential association between CBF and RDW between carriers and non-carriers of the APOE4 allele in distal and middle vascular flow territories. In the context of the different hypotheses about the disease this novel insight can further fuel our understanding of the mechanistical neurovascular alterations occurring during the disease’s trajectory. There is accumulating evidence for physiological and functional alterations occurring in APOE4 carriers at young or middle-age; cerebrovascular reactivity appears to be lower in APOE4 carriers,^69^ CBF has been found to be higher,^6^ hyper-activation has been observed^70^ and the blood brain barrier has been reported to be more leaky.^71^ In the present study there was a trend towards higher baseline CBF in APOE4 carriers. Though it is tempting to examine these findings through the prism of antagonistic pleiotropy (i.e. beneficial effects of the gene in younger ages), evidence of increased CBF and reduced cerebrovascular reactivity in APOE4 carriers points towards a baseline compensatory mechanism and neurovascular alterations occurring early in the disease’s trajectory. In the full PREVENT sample, we did not observe prominent hyperperfusion as was the case in the initial study sample ^7,13^ but there was a clear trend towards higher brain perfusion in the frontotemporal lobes. This difference in observations could be connected to one important limitation of our study, which was the difference in bolus duration between the different study sites as well as the varying FOV between and within sites. The bolus duration was the only imaging parameter that differed, however it is well-known that different inversion times impact CBF estimation.^72^ Following data collection this effect can only be addressed in post-processing. To that end, we specifically supplied to the *oxford_asl* algorithm the bolus duration, data were harmonized between sites and by taking bolus duration into account with COMBAT. The discrepancy between regional and voxel-wise analysis may be attributed to limited FOV coverage, which was not consistent between and within-sites. A final study limitation is that we did not consider independently different APOE genotypes.

Overall, we have shed new light on the pattern of underlying hyperperfusion in APOE4 carriers and investigated two different aspects: a) arterial transit time variations as captured by CoV and b) variations in the shape of erythrocytes as captured by RDW. Our analysis suggests that RDW was the measure that was differentially associated with CBF between APOE4 carriers and non-carriers, however, this was not explicatory of the hyperperfusion pattern. Rather, these associations were observed in areas which are more sensitive to ischemia, situated far from the feeding arteries. Hence, at this early stage whereby all participants in the study are cognitively asymptomatic, we have shown that the dominant risk gene for sporadic AD modulates differentially the association between cerebral perfusion and RDW, mainly in distal vascular territories. Further investigation is thus warranted on how hemodynamic and potentially hemorheological properties could be interacting with APOE4 carriership in impacting cerebrovascular health and physiology, especially in the absence of prominent differences both physiologically and structurally ^73,74^ between APOE4 carriers and non-carriers.

## Supplemental Material

sj-pdf-1-jcb-10.1177_0271678X231173587 - Supplemental material for Differential association of cerebral blood flow and anisocytosis in APOE ε4 carriers at midlife Click here for additional data file. Supplemental material, sj-pdf-1-jcb-10.1177_0271678X231173587 for Differential association of cerebral blood flow and anisocytosis in APOE ε4 carriers at midlife by Maria-Eleni Dounavi, Elijah Mak, Peter Swann, Audrey Low, Graciela Muniz-Terrera, Anna McKeever, Marianna Pope, Guy B Williams, Katie Wells, Brian Lawlor, Lorina Naci, Paresh Malhotra, Clare Mackay, Ivan Koychev, Karen Ritchie, Li Su, Craig W Ritchie and John T O’Brien: on behalf of the SVDs@target consortium in Journal of Cerebral Blood Flow & Metabolism
