# Supplementary material for: Differential association of cerebral blood flow and anisocytosis in APOE ε4 carriers at midlife
Source: J Cereb Blood Flow Metab. 2023 May 3;43(10):1672–84. doi: 10.1177/0271678X231173587 (PMC10581239; doi:10.1177/0271678X231173587)
Supplement: sj-pdf-1-jcb-10.1177_0271678X231173587 - Supplemental material for Differential association of cerebral blood flow and anisocytosis in APOE ε4 carriers at midlife [file sj-pdf-1-jcb-10.1177_0271678X231173587.pdf]

Supplementary Table 1: Robust linear regression results for the prediction of CBF and CoV using as predictors age, sex, education and APOE4. T and p-values are shown in the respective columns along with the overall model performance in the last column. A negative sign for APOE4 suggests that carriers have a lower value whereas a negative sign for sex that females have lower values for the examined metric. Abbreviations: ACA – anterior cerebral artery, APOE – apolipoprotein, CBF- cerebral blood flow, CoV – coefficient of variation, MCA- middle cerebral artery, PCA – posterior cerebral artery

	**age**	**educ**	**APOE4**	**sex**	**R^2; p**
**CBF**					
**ACAprox**	1.66; 0.10	-0.51; 0.61	1.61; 0.11	0.09; 0.93	0.016; 0.20
**ACAmid**	1.48; 0.14	-0.14; 0.89	1.29; 0.20	0.95; 0.34	0.012; 0.33
**ACAdist**	-0.26; 0.79	-0.25; 0.80	0.16; 0.87	**3.69; <0.01**	**0.037; 0.01**
**MCAprox**	**2.49; 0.01**	-0.22; 0.82	**2.54; 0.01**	-1.85; 0.07	**0.041; <0.01**
**MCAmid**	**2.81; 0.01**	-0.17; 0.87	1.76; 0.08	-0.99; 0.32	**0.031; 0.02**
**MCAdist**	1.50; 0.13	0.18; 0.85	0.52; 0.60	1.75; 0.08	0.014; 0.26
**PCAprox**	0.96; 0.34	0.81; 0.42	1.75; 0.08	1.46; 0.15	0.017; 0.17
**PCAmid**	-0.64; 0.52	1.34; 0.18	0.40; 0.69	1.41; 0.16	0.015; 0.24
**PCAdist**	**-1.93; 0.05**	1.25; 0.21	-0.30; 0.76	**4.20; <0.01**	**0.067; <0.01**
**CoV**					
**ACAprox**	**2.47; 0.01**	-0.82; 0.41	0.57; 0.57	**-4.34; <0.01**	**0.062; <0.01**
**ACAmid**	**2.15; 0.03**	-0.17; 0.87	0.93; 0.35	**-5.18; <0.01**	**0.064; <0.01**
**ACAdist**	**2.79; 0.01**	0.61; 0.54	1.29; 0.20	**-8.10; <0.01**	**0.132; <0.01**
**MCAprox**	**3.03; <0.01**	-1.07; 0.29	0.96; 0.34	**-5.55; <0.01**	**0.087 <0.01**
**MCAmid**	**3.14; <0.01**	0.07; 0.94	0.98; 0.33	**-8.67; <0.01**	**0.153; <0.01**
**MCAdist**	**3.58 <0.01**	0.19; 0.85	1.72; 0.09	**-10.63; <0.01**	**0.209; <0.01**
**PCAprox**	**3.49; <0.01**	0.19; 0.85	1.48; 0.14	**-4.88; <0.01**	**0.076; <0.01**

**PCAmid**	**3.04; <0.01**	0.39; 0.70	1.25; 0.21	**-6.20; <0.01**	**0.093; <0.01**
**PCAdist**	**3.84; <0.01**	0.03; 0.98	1.51; 0.13	**-9.35; <0.01**	**0.177; <0.01**

Supplementary figures

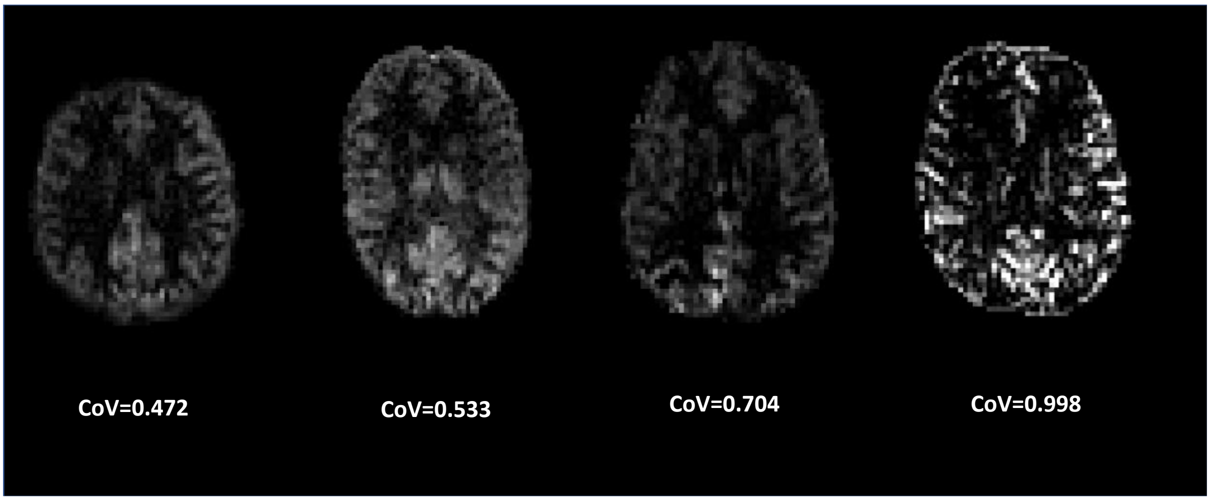

Supplementary Figure 1: Four examples in ASL native space of different spatial coefficients of variation (CoV)s. The shown maps are cerebral blood flow maps without partial volume correction.
